# Subgenome dominance and its evolutionary implications in crop domestication and breeding

**DOI:** 10.1093/hr/uhac090

**Published:** 2022-04-22

**Authors:** Zheng Wang, Jinghua Yang, Feng Cheng, Peirong Li, Xiaoyun Xin, Weihong Wang, Yangjun Yu, Deshuang Zhang, Xiuyun Zhao, Shuancang Yu, Fenglan Zhang, Yang Dong, Tongbing Su

**Affiliations:** Beijing Vegetable Research Center (BVRC), Beijing Academy of Agriculture and Forestry Science (BAAFS), Beijing 100097, China; National Engineering Research Center for Vegetables, Beijing 100097, China; Key Laboratory of Biology and Genetic Improvement of Horticultural Crops (North China), Ministry of Agriculture, Beijing 100097, China; Beijing Key Laboratory of Vegetable Germplasm Improvement, Beijing 100097, China; Laboratory of Germplasm Innovation and Molecular Breeding, Institute of Vegetable Science, Zhejiang University, Hangzhou 310058, China; Institute of Vegetables and Flowers, Chinese Academy of Agricultural Sciences, Beijing 100081, China; Beijing Vegetable Research Center (BVRC), Beijing Academy of Agriculture and Forestry Science (BAAFS), Beijing 100097, China; National Engineering Research Center for Vegetables, Beijing 100097, China; Key Laboratory of Biology and Genetic Improvement of Horticultural Crops (North China), Ministry of Agriculture, Beijing 100097, China; Beijing Key Laboratory of Vegetable Germplasm Improvement, Beijing 100097, China; Beijing Vegetable Research Center (BVRC), Beijing Academy of Agriculture and Forestry Science (BAAFS), Beijing 100097, China; National Engineering Research Center for Vegetables, Beijing 100097, China; Key Laboratory of Biology and Genetic Improvement of Horticultural Crops (North China), Ministry of Agriculture, Beijing 100097, China; Beijing Key Laboratory of Vegetable Germplasm Improvement, Beijing 100097, China; Beijing Vegetable Research Center (BVRC), Beijing Academy of Agriculture and Forestry Science (BAAFS), Beijing 100097, China; National Engineering Research Center for Vegetables, Beijing 100097, China; Key Laboratory of Biology and Genetic Improvement of Horticultural Crops (North China), Ministry of Agriculture, Beijing 100097, China; Beijing Key Laboratory of Vegetable Germplasm Improvement, Beijing 100097, China; Beijing Vegetable Research Center (BVRC), Beijing Academy of Agriculture and Forestry Science (BAAFS), Beijing 100097, China; National Engineering Research Center for Vegetables, Beijing 100097, China; Key Laboratory of Biology and Genetic Improvement of Horticultural Crops (North China), Ministry of Agriculture, Beijing 100097, China; Beijing Key Laboratory of Vegetable Germplasm Improvement, Beijing 100097, China; Beijing Vegetable Research Center (BVRC), Beijing Academy of Agriculture and Forestry Science (BAAFS), Beijing 100097, China; National Engineering Research Center for Vegetables, Beijing 100097, China; Key Laboratory of Biology and Genetic Improvement of Horticultural Crops (North China), Ministry of Agriculture, Beijing 100097, China; Beijing Key Laboratory of Vegetable Germplasm Improvement, Beijing 100097, China; Beijing Vegetable Research Center (BVRC), Beijing Academy of Agriculture and Forestry Science (BAAFS), Beijing 100097, China; National Engineering Research Center for Vegetables, Beijing 100097, China; Key Laboratory of Biology and Genetic Improvement of Horticultural Crops (North China), Ministry of Agriculture, Beijing 100097, China; Beijing Key Laboratory of Vegetable Germplasm Improvement, Beijing 100097, China; Beijing Vegetable Research Center (BVRC), Beijing Academy of Agriculture and Forestry Science (BAAFS), Beijing 100097, China; National Engineering Research Center for Vegetables, Beijing 100097, China; Key Laboratory of Biology and Genetic Improvement of Horticultural Crops (North China), Ministry of Agriculture, Beijing 100097, China; Beijing Key Laboratory of Vegetable Germplasm Improvement, Beijing 100097, China; Beijing Vegetable Research Center (BVRC), Beijing Academy of Agriculture and Forestry Science (BAAFS), Beijing 100097, China; National Engineering Research Center for Vegetables, Beijing 100097, China; Key Laboratory of Biology and Genetic Improvement of Horticultural Crops (North China), Ministry of Agriculture, Beijing 100097, China; Beijing Key Laboratory of Vegetable Germplasm Improvement, Beijing 100097, China; State Key Laboratory of Systematic and Evolutionary Botany, Institute of Botany, the Chinese Academy of Sciences, Beijing 100093, China; Beijing Vegetable Research Center (BVRC), Beijing Academy of Agriculture and Forestry Science (BAAFS), Beijing 100097, China; National Engineering Research Center for Vegetables, Beijing 100097, China; Key Laboratory of Biology and Genetic Improvement of Horticultural Crops (North China), Ministry of Agriculture, Beijing 100097, China; Beijing Key Laboratory of Vegetable Germplasm Improvement, Beijing 100097, China

## Abstract

Polyploidization or whole-genome duplication (WGD) is a well-known speciation and adaptation mechanism in angiosperms, while subgenome dominance is a crucial phenomenon in allopolyploids, established following polyploidization. The dominant subgenomes contribute more to genome evolution and homoeolog expression bias, both of which confer advantages for short-term phenotypic adaptation and long-term domestication. In this review, we firstly summarize the probable mechanistic basis for subgenome dominance, including the effects of genetic [transposon, genetic incompatibility, and homoeologous exchange (HE)], epigenetic (DNA methylation and histone modification), and developmental and environmental factors on this evolutionary process. We then move to *Brassica rapa*, a typical allopolyploid with subgenome dominance. Polyploidization provides the *B. rapa* genome not only with the genomic plasticity for adapting to changeable environments, but also an abundant genetic basis for morphological variation, making it a representative species for subgenome dominance studies. According to the ‘two-step theory’, *B. rapa* experienced genome fractionation twice during WGD, in which most of the genes responding to the environmental cues and phytohormones were over-retained, enhancing subgenome dominance and consequent adaption. More than this, the pangenome of 18 *B. rapa* accessions with different morphotypes recently constructed provides further evidence to reveal the impacts of polyploidization and subgenome dominance on intraspecific diversification in *B. rapa*. Above and beyond the fundamental understanding of WGD and subgenome dominance in *B. rapa* and other plants, however, it remains elusive why subgenome dominance has tissue- and spatiotemporal-specific features and could shuffle between homoeologous regions of different subgenomes by environments in allopolyploids. We lastly propose acceleration of the combined application of resynthesized allopolyploids, omics technology, and genome editing tools to deepen mechanistic investigations of subgenome dominance, both genetic and epigenetic, in a variety of species and environments. We believe that the implications of genomic and genetic basis of a variety of ecologically, evolutionarily, and agriculturally interesting traits coupled with subgenome dominance will be uncovered and aid in making new discoveries and crop breeding.

## Polyploidization and genome evolution

The apparent incongruence between haploid nuclear DNA contents (C-value) and organismal complexity, known as the C-value enigma, is prevalent across the eukaryotic tree of life [1, 2]. This paradox is particularly conspicuous in angiosperms, which exhibit a great diversity in genome size, with a 2400-fold difference between the smallest genome (63 Mb; *Genlisea margaretae*) and the largest genome (149 Gb; *Paris japonica*) [3, 4]. One factor responsible for this remarkable feature of genome complexity among angiosperms is recurrent lineage-specific whole-genome duplication (WGD, also referred to as polyploidization) and small-scale genome duplication events [5–7]. Polyploids commonly arise from accidental merging of unreduced gametes, in which cells or organisms acquire more than two sets of chromosomes [8]. Based on the parental genome status after polyploidization, polyploids are classified as neo- and palaeopolyploids. It is now widely accepted that the extant angiosperms evolved from palaeopolyploid ancestors with the genomic remnants of at least two ancient and independent WGDs [9–12]. In the process of WGD, selective expansion of transposable elements (TEs) contributed to the enormous differentiation of plant genome size [13]. Plant genomes tended to reduce in size as the result of TE loss and diploidization following WGDs due to adaptation to specific ecological niches of the plants [14].

**Figure 1:**
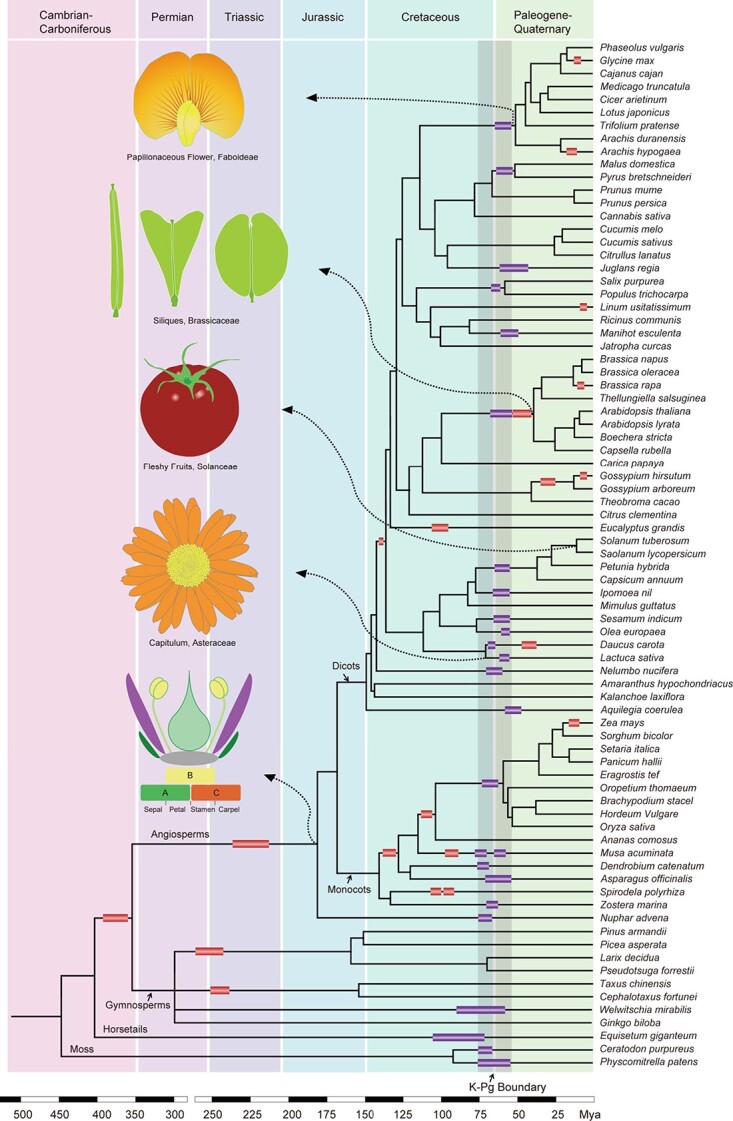
A phylogenetic tree of plants showing the association of WGDs with morphological innovations. A simplified phylogeny displaying the evolutionary relationship between representative plant species. Mapping of WGDs and key morphological innovations on the phylogeny is based on information from published data (Van de Peer *et al*., 2017; Cheng *et al*., 2018; [24, 36]). WGDs estimated to be between 55 and 75 million years old are shown in purple rectangular boxes, while others are shown in red rectangular boxes. The K–Pg boundary is indicated by a grey shaded area.

Studies have revealed that all current plant species have evolved from one or more palaeopolyploidizations, which might be associated with dramatic environmental changes. With the increasing availability of plant genome sequences, it is becoming clearer that a wave of polyploidization events apparently took place around the Cretaceous–Palaeogene (K–Pg) boundary, which marks an extinction event probably caused by a meteor strike that occurred 60–70 million years ago (Mya) [7, 15, 16] (Fig. 1). Polyploid plants exhibit increased adaptation to extreme environmental conditions compared with those of their diploid parents, allowing them to better survive these disastrous climates than their diploid progenitors. Alternatively, Freeling hypothesized that polyploids were merely by-products resulting from adaptive selection that occurred during long-term asexual reproduction underground or under water [17]. Therefore, it has been suggested that in plants polyploidization is important because of its close relationship with the diversification of plant species, novel gene functions [18, 19], the domestication of crops, and the formation of vital agronomic traits [18, 20–23].

## Polyploidization and evolutionary innovations in angiosperms

The evolutionary origin of flowers and fleshy fruits is considered a key morphological innovation that promoted the explosive radiation and propagation of angiosperms [24]. The developmental specificity of flower organs is determined by the combined action of MADS-box transcription factors, known as the ABC(DE)-model of floral organ identity [25, 26]. An ancient WGD that happened in the common ancestor of angiosperms was proposed to produce an array of genetic components that account for the origin of flowers (Fig. 1) [27]. Notably, lineage-specific duplication in the *AGAMOUS* gene clade resulted in the production of the C- and D-function lineages, which in turn specified the carpel and ovule identities, respectively [28]. It is likely that an ancient WGD, coupled with lineage-specific gene expansion, is tightly associated with floral organ elaboration in angiosperms. The evolutionary success of the sunflower family (Asteraceae) depends mainly on the development of their unique head-like inflorescence, termed the capitulum, which is linked to multiple documented WGDs [29, 30]. Fleshy fruits evolved independently to facilitate long-distance seed dispersal by attracting animals, and hence are considered to be an adaptive character that increased plant success and boosted adaptive radiation [31, 32]. For example, the origin of the key genetic regulators involved in fruit development and ripening in tomato (*Solanum lycopersicum*) was attributable to a WGD event dating from the K–Pg boundary [33]. Other evolutionary innovations, such as the origin of glucosinolates and subsequent structural elaborations in Brassicales, and the origin of rhizobial nodulations in Papilionoids, are all associated with lineage-specific WGDs [34–36]. These findings revealed that the polyploidizations contributed to gene function innovation, crop domestication, and establishment of important agronomic traits [34–36].

## Genome differentiation, subgenome dominance, and developmental mechanisms

Polyploidization is also categorized into allopolyploidization, in which a single nucleus is formed when the genomes of two different species hybridize, and autopolyploidization, representing genome duplication in the same species [11, 12]. In most allopolyploids, the sudden increase in duplicated genes is offset by gene fractionation, in which homoeologous subgenomes reciprocally lose genes or *cis*-regulatory elements, and in the due course the duplicated genes acquire single-copy status [37]. Dominance is a ubiquitous characteristic of great evolutionary importance [38], while in allopolyploids the phenomenon of subgenome dominance is crucial. Duplicated genes residing in the subgenome showing less fractionation tend to have higher expression levels and contribute more to morphological determination than those in the more fractionated subgenome [39]. Various taxa, such as maize [37], *Brassica* [39], cotton [40, 46], *Arabidopsis* [41, 45], *Tragopogon* [42], grasses [43], and wheat [44], were reported to possess subgenome dominance. In most of the above studies, subgenome dominance was discussed in terms of plant evolution and adaptation.

Researchers believe that mutation or deletion of upregulated gene copies tends to induce reduced fitness, which is likely to promote their retention by polyploids [39]. However, the genetic mechanism that generates and retains subgenome dominance remains obscure. Among these possible explanations, firstly, low TE abundance is believed to be associated with the biased gene expression in many allopolyploid species [47]. Genome stability is affected negatively by TE activation, whereas inactivation of TEs via methylation decreases the chance of transposon blooms but suppresses neighbouring gene expression. Consequently, genes in the TE-dense subgenome are less expressed than those in the TE-sparse subgenome, but not in all cases, such as when homoeologous genes from the recessive subgenome are expressed at a lower level than those from the dominant subgenome [39, 48]. Secondly, it is now generally believed that genetic incompatibility allows the evolution of dominance. Hybridization is the major origin of allopolyploidization; however, hybridization could disrupt complex regulatory networks [49]. Indeed, fitness may be reduced when highly dosage-sensitive constituents of complexes and pathways are disrupted [50]. Subgenome dominance has been reported to be affected by genetic incompatibilities in cells, such as the coordination of various metabolic, signalling, and regulatory networks, and as a result of merging contrasting diploid progenitor species’ genomes into a single nucleus. This might result in some pathways being controlled by one subgenome, while other pathways are controlled by the remaining subgenome [51]. This would result in phenotypic traits being partitioned to different subgenomes, such as in blueberry, cotton, and wheat [52, 53]. Thirdly, comparatively little is known about the extent to which chromatin dynamics affect subgenome dominance. In contrast to the stability and heritability of DNA methylation, more dynamism and plasticity are shown by higher-level chromatin modifications, including histone phosphorylation or acetylation. Recent studies of progenitor diploids and allopolyploid cotton revealed that extensive reorganization of domains associated with topology was correlated with alterations to methylation and chromatin status [54]. Gene expression and regulation are affected markedly by changes in DNA methylation and chromatin accessibility, which might be vital to establish subgenome dominance. Additionally, young allopolyploids are reported to undergo homoeologous exchanges (HEs), which can result in alterations to downstream phenotypes, genome-wide methylation patterns, and allele dosage, which might lead to genome stabilization and speciation events [55]. These findings were further supported by a report that the recently developed *Brassica napus* pangenome has highly variable levels of gene presence or absence among various cultivars, resulting from HEs involving genes associated with vital agronomic traits, such as chemical defence, disease resistance, and flowering time. Moreover, HEs display marked subgenome bias [56]. Consequently, more regions from one subgenome are substituted by regions from the other subgenome than the reverse situation, including in synthetic AADD wheat tetraploids, octoploid strawberry, and allopolyploid cotton [56–58]. In addition, HEs affect the expression level of a homoeolog according to the gene copy number [59].

## Roles of subgenome dominance in domestication and intraspecific diversification of *Brassica rapa* crops

Kagale *et al*. identified the polyploidization events and their corresponding times of occurrence in cruciferous species [60]. The α and β WGDs happened about 47 and 124 Mya, respectively, which was before the Brassicaceae family diversified, while another WGT occurred more recently (<23 Mya). Comparisons of the genomes of *B. rapa* and those of other cruciferous plants allowed the reconstruction of the three *B. rapa* subgenomes and the deduction of the diploid ancestral genome (2*n* = 14) that was present before the *Brassica* WGT event [61]. The different characteristics of the three *B. rapa* subgenomes prompted the authors to speculate that a tetraploid was formed by the merger of two ancestral genomes, and another hybridization with a third genome happened later. The 21 (3 × 7) ancestral chromosomes became reshuffled as a result of this two-step hexaploidization process, which, via re-diploidization, subsequently evolved into the present day *B. rapa* genome comprising 10 chromosomes [62]. The two-step polyploidization provided the *B. rapa* genomes with not only the genomic plasticity to adapt to changing environments, but also an abundant genetic basis for further morphological innovations and variations, which has enabled *B. rapa* to become one of the most diverse species among the angiosperms.

According to the ‘two-step theory’, the *B. rapa* genome experienced genome fractionation twice after the WGD event, subsequently forming three subgenomes [39]. The two-round genome fractionation resulted in extensive gene loss in the three subgenomes; however, most of genes responding to the environmental cues and phytohormones were over-retained during this process, which further enhanced the adaptation and varied morphotypes of *B. rapa* [62]. *B. rapa*’s close relationship to *Arabidopsis thaliana* offers a good opportunity to study the diversification of the three subgenomes. Further analysis revealed the least fractionated subgenome (LF) retained a higher gene density than the moderately fractionated subgenome (MF1) and the most fractionated subgenome (MF2) [62]. Paralogous genes from the LF subgenome showed a dominant pattern over those from the MF1 and MF2 subgenomes, indicating that the subgenome dominance was caused by the biased fractionation after the WGDs [39]. In addition, the dominant expression status of pairwise syntenic paralogue genes was stable in different organs and different *B. rapa* varieties, and this dominant pattern correlated negatively with the biased distribution of TEs, which is consistent with the aforementioned pattern. However, TEs are not the only factors involved in the emergence of the dominant subgenome of *B. rapa*. Interestingly, 24-nucleotide small RNAs preferentially targeted to the TEs of the MF subgenomes subsequently resulted in TE methylation, which inhibited the expression of downstream genes [63].

**Figure 2:**
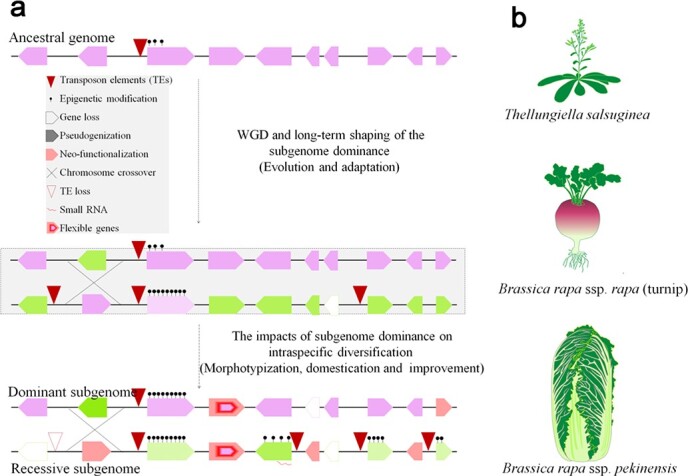
Subgenome dominance and its evolutionary implications in plants. **a** Establishment of subgenome dominance and its evolutionary implications in crop domestication and intraspecific diversification. Subgenome dominance was initially shaped and influenced by TE density, epigenetic modification, HE, and other unexplored factors when two distinct subgenomes merge. (Dotted box) To better understand the effects of subgenome dominance on intraspecific diversification, we assumed an intermediate status during the shaping of subgenome dominance, which actually does not exist during evolution. **b** Polyploidization and subgenome dominance constitute the fundamental driving force for evolution and morphotypization of *B. rapa.*

Quantitative trait locus (QTL) mapping and *de novo* assemblies of different subspecies provide evidence and a genetic basis for the evolution and diversification of *B. rapa* crops [62, 64–68]. However, unique populations and few reference genomes have limited the identification of the structure variation and copy number variation of *B. rapa* and cannot resolve the genetic diversity of such a diverse species. Recently, a high-quality graph-based pangenome was constructed, providing important genetic information and shedding new light on *B. rapa* evolution and domestication [69]. The 18 genome accessions used in the *de novo* assembly represent most of the morphotypes of *B. rapa*, including the turnip, heading Chinese cabbage, non-heading pak choi, oilseed, sarsons, broccolieto (keto), and mizuna. Compared with the reference Chiifu (a heading Chinese cabbage) genome, the other 17 genomes appeared to show that 15.14–37.39% of the sequences of each genome had no synteny with the reference genome, indicating extensive genomic variation among different subspecies, which is in line with the fact that different *B. rapa* subspecies have distinct morphological characteristics [70]. More importantly, Cai *et al*. [69] offered a novel insight into the evolution and intraspecific diversification of *B. rapa*, which goes further than the two-step theory. In the process of diversification, *B. rapa* developed into highly diverse morphotypes, which provide a powerful reference to investigate the effects of subgenome dominance on intraspecific diversification (Fig. 2). Cai *et al*. provided further evidence to support subgenome dominance at the intraspecific level by defining the conserved syntenic genes (CSGs) and flexible syntenic genes (FSGs) in *B. rapa* crops. There was a significantly lower proportion of FSGs in the LF subgenome than in the MF1 and MF2 subgenomes, indicating that the expansion of LF subgenome dominance might, at least partially, be explain by intraspecific diversification-related gene flexibility. Interestingly, FSGs tend to accumulate more non-synonymous mutations, structure variations, large-effect mutations, and long terminal repeat retrotransposons, and thus could be tightly associated with the morphological diversification and domestication of *B. rapa*. Furthermore, an inferred ancestral genome was constructed to study the fractionation and subgenome dominance during intraspecific diversification. Consistent with the lower gene loss rate observed in the LF subgenome in different *Brassica* crops [62], the LF subgenome retained more genes from the inferred *B. rapa* ancestral pangenome and showed a lower fractionation rate compared with the MF subgenomes. However, the ratio of FSGs was significantly higher in the MF subgenomes; this further increased LF subgenome dominance during intraspecific diversification.

## Future perspectives and application of subgenome dominance knowledge in crop breeding

Subgenome dominance does not mean absolute dominance and expression bias. First, studies showing that local regions favouring homoeologs of one subgenome dominate the others are usually observed in many plants, such as wheat, *B. napus* [71] and cotton [72], but the global expression was not biased towards one specific subgenome. In other words, the submissive subgenome still encompasses certain genes that are more highly expressed than the homoeologs in the dominant subgenome. Next, in some cases the homoeologs in the submissive subgenome are more highly expressed in certain spatial and temporal contexts. Take the tetraploid blueberry, for example: while one subgenome has higher expression in nearly all tested tissues and developmental phases, the other subgenome is highly expressed during fruit development [52]. Similar results were observed in allotetraploid cotton [53]. We thus deduced that the submissive subgenome may fit better in a particular developmental stage, and thus selection may aid it to be dominantly expressed in certain spatiotemporal contexts. Additionally, many polyploids were derived from hybridization, whereas it is reported that ecological environments during hybridization affect the arising of subgenome dominance and lead to polyploid adaptability. We thus assume that the environmental context could influence which subgenome becomes dominant in certain polyploids. On the other hand, it is reported that abiotic stress can adjust expression patterns in synthetic polyploid cotton, while natural polyploids remain largely unchanged [73]. All the above findings revealed that pre-existing differences between parental genomes could influence subgenome bias and further show that, if subgenome dominance can be predicted in certain hybrids and/or polyploid species, it will be possible to decide which will be of great use for breeding.

Further to the above discussion, it is intriguing that the bias in TE density and DNA methylation, as well as chromatin modification and HEs, do not always result in biased subgenome-wide gene expression; therefore, it is still an open question as to how these processes, working individually or together, affect subgenome dominance. Thus, resynthesized and corresponding natural allopolyploids, and their polyploid progenitors, represent a helpful system to investigate subgenome dominance establishment and escalation. Pre-existing differences between parental genomes, in terms of TE density, DNA methylation, and consequent gene expression, affect the dynamics of allopolyploid subgenomes [74, 75]. The tendency in some species for parental differences to be mimicked in subgenome dominance patterns has been named the ‘parental legacy’ [74]. However, it remains unclear to what extent the development of subgenome expression bias occurs because of pre-existing diploid progenitor features or results from non-recurrent and independent events during the formation of polyploids. On the other hand, we should bear in mind the clear differences between hybrids and polyploids. For example, hybrids have only one set of chromosomes from each parental progenitor, and this heterozygosity makes hybrids unstable; in contrast, polyploid subgenomes comprise two sets of homologous chromosomes from each parent, which are inherited stably in each generation. In addition, Han *et al*. demonstrated that the chromosomes of synthetic cotton and natural cotton tetraploids associate together dependent on their parent of origin [76], which prompted us to hypothesize that chromosomes do not rearrange themselves randomly. Therefore, there is an urgent need to take advantage of sophisticated techniques, such as the Hi-C chromosome conformation capture application for DNA–DNA interaction and single cell RNA-sequencing as back-up for cell-specific gene expression, to stimulate post-polyploidy chromosomal interaction and neo-functional annotation analyses. Last but not least, we believe that histone modification and chromatin accessibility profiling could be used in polyploidy lineages to gain a better understanding of their effect on subgenome dominance establishment and evolution.

Studies on the functional divergence of homoeologs upon polyploidization revealed that the biased expression of homoeologs is associated with genome-wide selection, thus implying that transcriptional subgenome dominance enables trait selection [77, 78]. As seen in *B. rapa*, genetic studies and transcription analyses have identified certain QTLs and candidate regulators involved in the morphological variations of different *B. rapa* species [79–84]. As summarized in Table 1, we observed significant impacts of subgenome dominance on *B. rapa* diversification, the formation of morphotypes, domestication, and crop improvement, as indicated by the fact that genes of the LF subgenome were more likely to be selected, either naturally or artificially, during all the above evolutionary events. Besides, in allopolyploid *Brassica juncea*, homoeolog expression dominance has also aided the selection of genes related to lipid and glucosinolate metabolism in subvarieties used for oil production or as vegetables [78]. In addition, in allopolyploid wheat, directional selection had different effects on duplicated homoeologs, which resulted in contrasting variation patterns and inter-variant associations among wheat genomes [77]. Thus, when considering the domestication and improvement of crops, the identification of bias-expressed functional homoeologs is important in order to determine or engineer the genetic basis or checkpoint for interesting traits [85, 86]. These observations on the differential expression of homoeologs suggested that polyploid crop breeding programmes could be improved by focusing on that subset of genes showing subgenome dominance, to enhance both the response to selection and the acquisition of mechanistic insights. Furthermore, it was revealed that parental legacy, depending on the differences in the expression or epigenetic modifications of gene pairs in the parents or progenitors, resulted in dominance in the remodelling of homoeolog expression bias and asymmetrical epigenetic modifications in *Brassica* and other crops [66]. Therefore, we believe that determining the genetic and epigenetic regulatory mechanisms of the differential expression of homoeologs, as well as the fact that the advantages of heterosis are encompassed by polyploidy-based breeding, will facilitate the *de novo* domestication or improvement of newly synthesized allopolyploids, which could be developed into new crops to strengthen food security. Actually, with recent advances in genetic engineering technology, *de novo* domestication of newly synthesized allopolyploids has attracted the attention of many cutting-edge scientists [87, 88].

**Table 1:** Summary of candidate genes related to important agricultural traits in *Brassica rapa*

**Trait**	**Gene**	**Name**	**Chromosome**	**Position**	**Subgenome**	**Identification method**	**Reference**
**Heading**	*BrPIN3.3*	BraA07g030650.3C	A07	21 870 249	LF	Selection sweep	69
	*BrMYB3.3*	BraA07g029180.3C	A07	21 145 989	/	Selection sweep	69
	*BrFL5.1*	BraA01g019170.3C	A01	10 320 303	LF	Selection sweep	69
	*BrSAL4.2*	BraA01g025930.3C	A01	15 373 141	MF1	Selection sweep	69
	*BrARF3.1*	BraA04g024390.3C	A04	17 723 081	LF	Selection sweep	63
	*BrARF4.1*	BraA10g018230.3C	A10	13 535 905	LF	Selection sweep	63
	*BrKAN2.1*	BraA09g032840.3C	A09	25 471 282	LF	Selection sweep	63
	*BrKAN2.3*	BraA05g023490.3C	A05	17 380 156	MF2	Selection sweep	63
	*BrBRX.1*	BraA09g033250.3C	A09	25 879 277	LF	Selection sweep	63
	*BrBRX.2*	BraA08g009040.3C	A08	7 993 035	MF1	Selection sweep	63
	*BrSPL9*	BraA05g002720.3C	A05	1 497 805	LF	Homologous cloning	Wang *et al*., 2013
	*BrKS1*	BraA07g042410.3C	A07	28 393 881	LF	MutMap analysis	82
**Tuber formation**	*BrSTP1.1*	BraA06g007950.3C	A06	4 363 461	LF	Selection sweep	63
	*BrSTP1.3*	BraA09g061400.3C	A09	42 842 596	MF2	Selection sweep	63
	*BrEXPB3.2*	BraA03g054290.3C	A03	28 172 489	MF1	Selection sweep	63
	*BrFR7.1*	BraA07g026700.3C	A07	20 256 386	MF2	QTL; MutMap analysis	84
**Flowering**	*BrFLC1*	BraA10g027720.3C	A10	18 122 666	LF	QTL; domestication	Yuan *et al*., 2009
	*BrFLC2*	BraA02g003340.3C	A02	1 616 321	MF2	QTL; domestication	Xiao *et al*., 2016
	*BrFLC5*	BraA03g015950.3C	A03	7 336 775	MF2	QTL	Xi *et al*., 2018
	*BrVIN3*	BraA06g040160.3C	A06	26 686 911	MF2	QTL; selection sweep	Su *et al*., 2018
	*BrFT1*	BraA02g016700.3C	A02	8 897 950	MF1	QTL; selection sweep	Su *et al*., 2018
	*BrCLF1*	BraA04g017190.3C	A04	13 126 173	MF1	EMS; Map-based cloning	Huang *et al*., 2020
	*BrSDG8*	BraA07g040740.3C	A07	27 592 362	MF1	EMS; Map-based Cloning	Fu *et al*., 2020
**Trichome formation**	*BrpHL1a*	BraA06g037290.3C	A06	24 917 368	LF	GWAS;QTL	Zhang *et al*., 2018
**Leaf colour**	*BrChlH*	BraA03g005840.3C	A03	2 557 478	MF1	EMS; MutMap analysis	Fu *et al*., 2019
	*Brnym1*	BraA03g050600.3C	A03	25 985 593	MF1	EMS; MutMap analysis	Wang *et al*., 2020
	*BrCRTISO*	BraA09g063710.3C	A09	43 923 664	MF2	Map-based cloning	Su *et al*., 2015
	*Brhisn2*	BraA05g023920.3C	A05	17 771 508	MF2	Map-based cloning	Su *et al*., 2021
	*Brmyb2*	BraA07g032100.3C	A07	23 201 361	LF	Map-based cloning	He *et al*., 2020
**Disease resistance**	*BrCRT2*	BraA06g006120.3C	A06	3 535 037	LF	GWAS; map-based cloning	Su *et al*., 2019

In the present perspective, we discuss recent findings regarding the mechanisms underlying subgenome dominance in hybrids and allopolyploids, which have profound implications for evolutionary, ecological, and agricultural research. Therefore, there is a requirement to harness advances in various omics technologies to develop a research pipeline to provide a deeper understanding of polyploidization and subgenome dominance and their mechanistic interdependencies. Moreover, it is now possible to use genome editing tools directly to induce mutation by targeting the key genes involved in TE insertion, DNA methylation, histone modification, and HEs associated with subgenome dominance and biased expressions, which will facilitate mechanistic investigations and advanced crop breeding.

## Acknowledgements

This work was supported by grants from the Natural Science Foundation of China (32002057, 32172557, 32170227), the Innovation and Capacity-Building Project of BAAFS (KJCX20210427), “Young Talent Award” of Beijing Agricultural and forestry Science and Science Innovation Program (KYCX202001-07).

## Author contributions

T.B.S., Y.D., Z.W., J.H.Y., and F.C. wrote the paper. T.B.S. and Y.D. carried out the analyses and drew the figures. P.R.L., X.Y.X., and W.H.W. revised the paper. All authors discussed the results and commented on the manuscript.

## Data availability

The data that support the findings of this study are available from the corresponding author upon reasonable request.

## Conflict of interest

The authors declared no conflicts of interest with respect to the authorship and/or publication of this article.
